# Vibrational spectroscopic profiling of biomolecular interactions between oak powdery mildew and oak leaves†

**DOI:** 10.1039/d3sm01392h

**Published:** 2024-01-03

**Authors:** Kieran R. Clark, Pola Goldberg Oppenheimer

**Affiliations:** a School of Chemical Engineering, Advanced Nanomaterials Structures and Applications Laboratories, College of Engineering and Physical Sciences, University of Birmingham Edgbaston Birmingham B15 2TT UK GoldberP@bham.ac.uk; b Birmingham Institute of Forest Research, University of Birmingham Edgbaston Birmingham B15 2TT UK; c Healthcare Technologies Institute, Institute of Translational Medicine Mindelsohn Way Birmingham B15 2TH UK

## Abstract

Oak powdery mildew, caused by the biotrophic fungus *Erysiphe alphitoides*, is a prevalent disease affecting oak trees, such as English oak (*Quercus robur*). While mature oak populations are generally less susceptible to this disease, it can endanger young oak seedlings and new leaves on mature trees. Although disruptions of photosynthate and carbohydrate translocation have been observed, accurately detecting and understanding the specific biomolecular interactions between the fungus and the leaves of oak trees is currently lacking. Herein, *via* hybrid Raman spectroscopy combined with an advanced artificial neural network algorithm, the underpinning biomolecular interactions between biological soft matter, *i.e.*, *Quercus robur* leaves and *Erysiphe alphitoides*, are investigated and profiled, generating a spectral library and shedding light on the changes induced by fungal infection and the tree's defence response. The adaxial surfaces of oak leaves are categorised based on either the presence or absence of *Erysiphe alphitoides* mildew and further distinguishing between covered or not covered infected leaf tissues, yielding three disease classes including healthy controls, non-mildew covered and mildew-covered. By analysing spectral changes between each disease category per tissue type, we identified important biomolecular interactions including disruption of chlorophyll in the non-vein and venule tissues, pathogen-induced degradation of cellulose and pectin and tree-initiated lignification of cell walls in response, amongst others, in lateral vein and mid-vein tissues. *Via* our developed computational algorithm, the underlying biomolecular differences between classes were identified and allowed accurate and rapid classification of disease with high accuracy of 69.6% for non-vein, 73.5% for venule, 82.1% for lateral vein and 85.6% for mid-vein tissues. Interfacial wetting differences between non-mildew covered and mildew-covered tissue were further analysed on the surfaces of non-vein and venule tissue. The overall results demonstrated the ability of Raman spectroscopy, combined with advanced AI, to act as a powerful and specific tool to probe foliar interactions between forest pathogens and host trees with the simultaneous potential to probe and catalogue molecular interactions between biological soft matter, paving the way for exploring similar relations in broader forest tree-pathogen systems.

## Abstract 2

Probing biomolecular interactions between a forest foliar pathogen, *Erysiphe alphitoides* and English oak (*Quercus robur*) leaves *via* hybrid Raman spectroscopy and accompanying analytical techniques.

## Graphical abstract

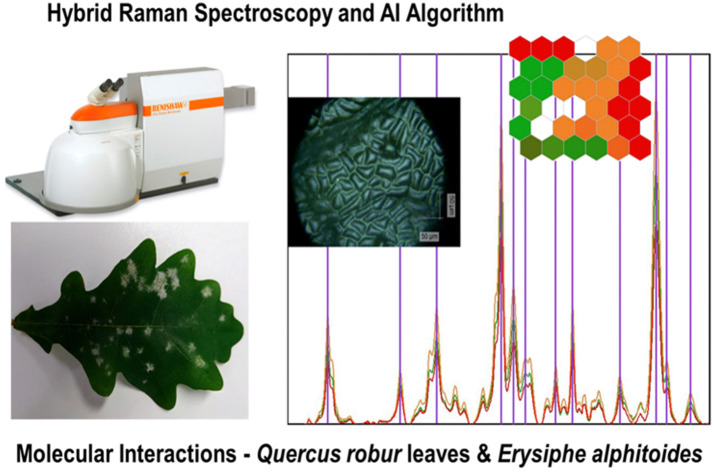

## Introduction

Powdery mildew of English oak (*Quercus robur*), oak PM, caused by the filamentous fungal pathogen *Erysiphe alphitoides* (formerly *Microsphaera alphitoides*), is an endemic foliar tree disease, which not only affects English oak trees, but also other *Quercus* species with diminishing severities.^1^ Whilst oak PM does affect young leaves of mature trees more than existing leaves of the latter,^2^ the concerning factor is the impact the disease has on young leaves growing on oak seedlings.^3^ The fungus *E. alphitoides* presents as a white mycelial layer on the adaxial surfaces of the leaves, which greatly affects seedling growth and often causes premature death, impacting the rejuvenation of forests.^4^ For mature trees, whilst having a higher resistance,^2^ repeated infections over numerous years have been shown to substantially affect their growth.^5^ Severe powdery mildew infections result in denser or higher percentage coverage of the leaf, which can be quantitatively assessed *via* either machine learning-based or pixel analysis-based image processing techniques and typically further requiring a spectroscopic-based method to quantify how much of a given leaf is covered with mildew.^6–13^ During mycelial coverage, the net rates of photosynthesis and transpiration of the affected leaves are reduced,^14^ leading to a decrease in growth and net carbon uptake during the growing season.^15^ The haustoria, specialised fungal cellular structures designed to siphon nutrients away from the leaf,^16^ also disrupt the translocation of photosynthates from the leaf into the tree. Active translocating and repurposing of certain carbohydrates such as sucrose also occurs.^17^ These disruptions of key processes for leaf growth are why oak PM is so deadly for younger leaves and seedlings, compared to mature trees. Outside of the global interactions of oak PM on oak trees, there is a lack of knowledge regarding the specific biomolecular changes occurring within oak leaves either caused by the *E. alphitoides* pathogen or by the *Q. robur* tree in response to the presence of pathogen. These leaf-specific changes are collectively referred to as “oak PM-oak leaf interactions” hereafter. To tackle the complex issue of filamentous pathogens in leaves and to further understand these diseases, there is an unmet need to rapidly probe the biomolecular changes to leaf structure, determine the mechanisms of infection and to identify any defence mechanisms. Furthermore, challenges posited by Tang *et al.*^18^ include the precise cataloguing of biomolecular biological soft matter interactions referring to microorganisms such as the *E. alphitoides* fungi or whole tissue of plants such as the *Q. robur* leaves. For such, the application of Raman spectroscopy would concurrently enable progress towards a more holistic understanding of biomolecular interactions between biological soft matter, vibrational spectroscopy-based biomolecular interaction cataloguing and subsequent understanding of oak PM-oak leaf interactions, ultimately having the potential to be revolutionary for forest pathology in the longer term.

Raman spectroscopy (RS) is a non-destructive, non-invasive analytical technique, which uses the inelastic scattering of photons from a sample to probe the biomolecular structure of the measured material.^19^ This allows accurate and rapid measurement of the chemical fingerprint of biological samples including for instance, foliar pathogens *e.g.*, rose rosette disease on rose leaves,^19^ tomato yellow leaf curl Sardinia virus and tomato spotted wilt virus on tomato leaves,^20^ citrus greening on orange and grapefruit leaves,^21,22^*Liberibacter* disease on tomato leaves,^23^*Abutilon* mosaic virus on leaves from the *Abutilon* species^24^ as well as fungal infections of cereal crops.^25^ Another approach to understand forest pathogen–host interactions involves considering the physicochemical interactions on the surface of the leaf host. This incorporates a wettability study^26,27^ whereby, drops are deposited onto the leaf's surface with a subsequent measurement of static contact angle between the solution and the surface at the point they meet.^28^ Two primary factors affecting the wettability of leaves include the surface chemistry of the cuticular and epicuticular layers, primarily formed of hydrophobic aliphatic chains of the order of C_20_–C_35_^29^ and the surface roughness, usually affected by the three-dimensional ultrastructure of the epicuticular waxes.^27,30^ The wettability study thus allows an assessment of changes to these factors throughout infection of the forest pathogen on the tree host's tissue.

Herein, the vibrational technique RS constitutes a promising tool to probe forest pathogen–host interactions *via* sensitive and accurate detection of a fungal forest pathogen on a leaf. Amongst the optical techniques, RS offers the richest and most sensitive spectroscopic discrimination wherein, Raman spectrum defines a unique chemical fingerprint that is determined by the underlying molecular constituents of the sample at the time of measurement. RS procedure has been developed, optimised and applied to profile the biomolecular interactions between oak powdery mildew and oak leaves, generating unique spectral fingerprints. Since, during an infection, oak PM-oak leaf interactions result in biomolecular changes to the leaf, which provide clues regarding the mechanism of infection and the tree-initiated defence response. We have examined the biomolecular interactions of the fungal pathogen *E. alphitoides* on *Q. robur* leaves during oak PM infection. The generated Raman spectral signatures were subsequently classified *via* our novel advanced artificial neural network (ANN) algorithm, the self-optimising Kohonen index network (SKiNET). SKiNET acts as an inherent decision support tool, by providing a framework for multivariate analysis, which simultaneously provides dimensionality reduction, feature extraction and multiclass classification. SKiNET is an ANN architecture based on self-organising maps (SOMs), whereby a spectrum is chosen at random from the training pool and matched with a neuron, which has previously corresponded to spectra like the chosen one. This neuron is more likely to further match with similar identified spectra and neurons close to the matched neuron are also likely to match with similar spectra, albeit to a lesser degree. These neurons are subsequently grouped, and colour coordinated according to the class the chosen spectrum belongs to and displayed as coloured hexagons, wherein each represents a neuron. If the classes are spectrally similar, this yields a hexagon of mixed colours, showing the relative proportion of matches from each class. The coloured neurons are then exhibited as a visually intuitive 2D array of hexagons, *i.e.*, the SOM, displaying class distribution, levels of intra-class mixing and degree of separation between classes. The SOM discriminant index (SOMDI) component of the ANN algorithm highlights the most dominant spectral features, which directly translate to the biomolecular differences of the highest importance in producing the respective SOM clustering. Our generated SOM and SOMDI classifications have been validated *via* a wettability study, identifying changes to hydrophobicity between healthy and oak PM infected oak leaves.

Overall, by integrating Raman spectroscopy with SKiNET, we demonstrate a valuable, non-destructive tool for the probing of tree pathogen–host tissue interactions, revealing new insights into the infections of *Q. robur* leaves by the *E. alphitoides* fungus. The combination of this spectroscopic technique with advanced AI paves the way for exploring similar interactions in various forest tree-pathogen systems and other biological soft matter.

## Materials and methods

### Plant samples acquisition

*Q. robur* leaves were collected from oak seedlings at two sites at the University of Birmingham, UK (52° 26′ 58.92′′ N, 1° 56′ 8.916′′ W), with healthy leaves from a greenhouse facility (Site 1) and infected leaves from a laboratory (Site 2). Site 1 experienced daily temperature variations of between 15–25 °C whilst Site 2 had a range of 10–30 °C. Leaves which are 6–12 months old, estimated from time of first emergence, were collected randomly from healthy and oak PM affected *Q. robur* seedlings, determined following a visual inspection of the leaves, by cutting at the base of the petiole. Leaves which showed signs of insect or physical damage were discarded due to the possibility of unknown factors. Collected leaves were placed into a sterile Petri dish, sealed to prevent contamination during transport and subsequently were analysed *via* Raman spectroscopy, ultraviolet-visible (UV-Vis) spectrophotometry, scanning electron microscopy (SEM) and contact angle measurements within an hour of collection.

### Raman spectroscopy

**Fig. 1:**
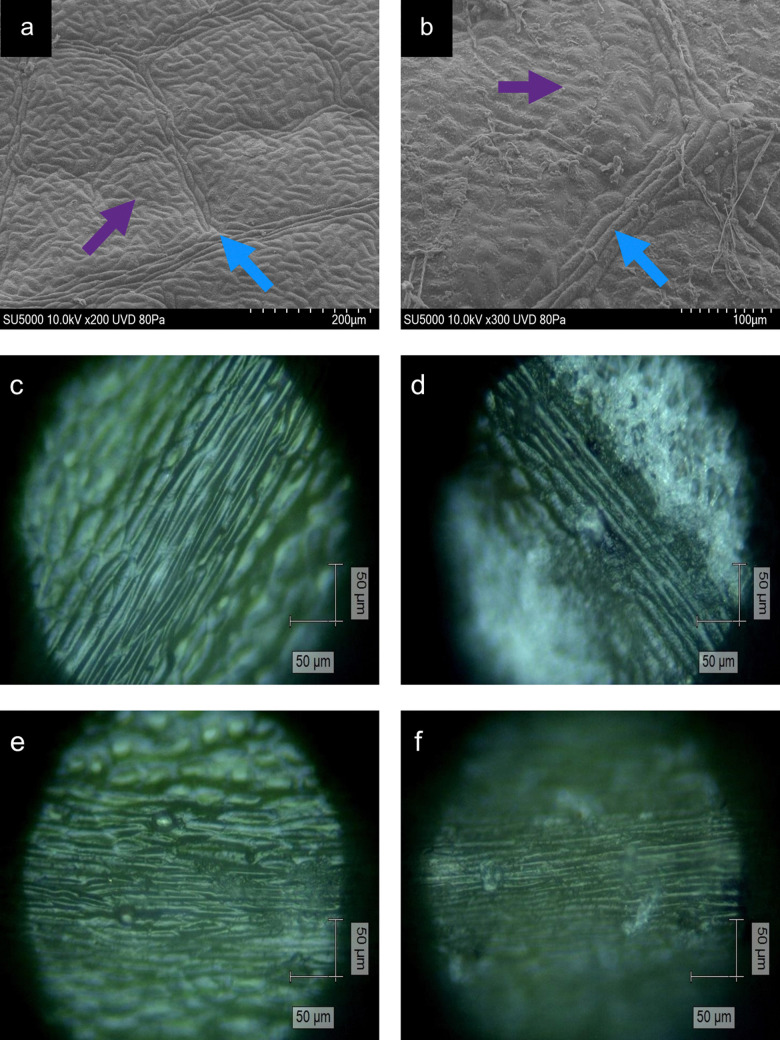
SEM images of non-vein (200× magnification) and venule tissues (300× magnification) (a) and (b), which were either healthy (a) or infected with *E. alphitoides* (b), where the purple arrows denote non-vein tissues whilst blue denotes venule tissues. Optical microscopy images at ×20 magnification of *Q. robur* samples of (c) and (d) lateral vein and (e) and (f) mid-vein tissues, which were either healthy (c) and (e) or infected with *E. alphitoides* (d) and (f).

Following the collection of the oak leaves, Raman spectra were acquired *via* an InVia Qontor Renishaw Raman Spectrometer (Renishaw Plc.), equipped with an 830 nm, 500 mW laser at ×20 magnification. Four distinct tissue types were identified on the adaxial surface of the leaf: non-vein (NV), venule (Ven), lateral vein (LV) and mid-vein (MV). The leaves were assigned a disease class according to a visual examination and history of the collected leaf. Leaves collected from Site 1 were assumed to be healthy (HOL), whereas infected leaves collected from Site 2 were assigned to be mildew-covered (MCOL) or non-mildew covered (NMCOL). Both MCOL and NMCOL measurements were acquired from the same oak leaf, dependant on the PM coverage (Fig. 1). Areas of tissue were measured per leaf using 4 point, 2 × 2 map scans with a 50 μm increment between points, for a total of 70–75 spectra per disease class. To produce optimal signal-to-noise ratio spectra, the experimental parameters for each tissue type and disease class were determined to be 60 s acquisition time, 6.16 mW power and 5 acquisitions. Following spectral profiling, the measured area was visually examined for signs of photo- or thermo-degradation of the leaf surface, with no visible signs for all the examined leaves.

### UV-Vis spectrophotometry

Strips of HOL, NMCOL and MCOL blade *ca.* 3 mm in width and 27.5 mg in mass were cut from fresh oak leaf samples. Chlorophyll was extracted from this tissue with *ca.* 2 mL of 80% DMSO in a glass tube, until the strips were submerged, following the procedure by Shinano *et al.*^31^ The glass tubes were then heated to 65 °C and kept at this temperature for two hours whilst being shaken at regular intervals. Absorbances at 648 nm and 665 nm (*A*_648_ and *A*_665_ respectively) were determined by a Cary 60 UV-Vis spectrophotometer (Agilent), blanked with 80% DMSO. The concentrations of chlorophyll *a* and *b* (in μg mL^−1^) were determined using the following equations:^32^ chlorophyll *a* = 14.85*A*_665_ − 5.14*A*_648_ and chlorophyll *b* = 25.48*A*_648_ − 7.36*A*_665_.

### Scanning electron microscopy

Areas of NV and Ven tissue on healthy and infected leaves were imaged using a SU5000 Schottky Field Emission Scanning Electron Microscope (Hitachi). Samples were imaged by being placed in a low-pressure mode (80 Pa), using an accelerating voltage of 10 kV, a working distance of 21–23 mm and at a tilt of between 51–53°.

### Contact angle measurements and wettability

Three different areas of 6–12-month-old HOL, NMCOL and MCOL samples collected in September were subjected to contact angle measurements using a Theta Optical Tensiometer (Biolin Scientific). Static contact angles were measured on drops of water with a volume range of 2–6 μL. A circular fitting algorithm with no baseline tilt was employed to determine the static contact angles and drops were imaged using the Attension software (Biolin Scientific). Wettability was subsequently classed according to measured contact angle (Table 1)^26^ and work of adhesion was calculated using the Young-Duprè equation, *i.e.*, *W*_SL_ = *γ*_L_(cos *θ* + 1) where, *W*_SL_ is the work of adhesion, *γ*_L_ is the surface tension of the water on the solid surface (71.99 mN m^−1^ at 25 °C) and *θ* is the contact angle on the surface.^33^

**Table 1:** Classes of wettability from measured contact angles

Contact angle (°)	Wettability class
0 < *θ* < 40	Super-hydrophilic
40 < *θ* < 90	Highly wettable
90 < *θ* < 110	Wettable
110 < *θ* < 130	Non-wettable
130 < *θ* < 150	Highly non-wettable
*θ* >150	Super-hydrophobic

### Multivariate data analysis

Wire 5.3 (Renishaw Plc.) software was used to process the collected spectra, subtracting the baseline and removing cosmic rays. Spectra from each collected disease class were averaged to produce a single spectrum per tissue type and per disease class. The spectra were then plotted using Origin 2023 (OriginLab Corporation). Once baseline subtracted, the intensities of the peaks at 898, 917, 1158, 1266, 1288 and 1526 cm^−1^ were determined. Ratios of intensities were calculated as follows: 898/917 = *I*(898 cm^−1^)/*I*(917 cm^−1^); 1267/1288 = *I*(1267 cm^−1^)/*I*(1288 cm^−1^) and 1526/1158 = *I*(1526 cm^−1^)/*I*(1158 cm^−1^), where *I*(*x* cm^−1^) refers to the intensity at *x* cm^−1^. Average ranges of intensities were calculated as follows, 
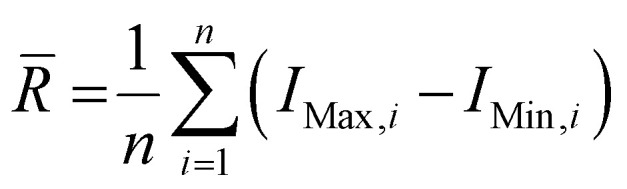
, where *R̄* is the average range, *n* is the total number of spectra, *I*_Max,*i*_ is the maximum intensity for the *i*-th spectrum and *I*_Min,*i*_ is the minimum intensity for the *i*-th spectrum. The coefficients of variation (CV) were calculated as 
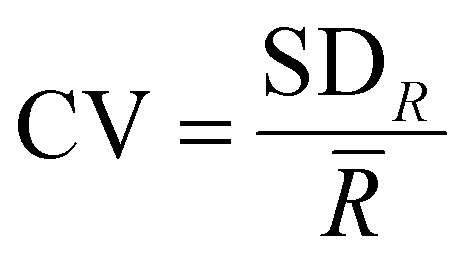
, where SD_*R*_ is the standard deviation of the ranges. The ratios of intensities, average ranges of intensities and chlorophyll concentrations are given as mean ± standard deviation. Due to the non-normal distribution of the average range of intensities, the intensity ratios, the contact angles, the adhesion data and the chlorophyll concentrations, Mann–Whitney *U* analysis was carried out over these ratios and *p* values <0.05 were determined as being statistically significant. Statistical analysis was performed using Origin 2023 (OriginLab Corporation). The baseline subtracted spectra were normalised using the Wire 5.3 software, for the subsequent SKiNET analysis to classify data. Multi-variate analysis was performed using SKiNET, described in detail in ref. 34 and 35 (Supplementary Information S1, ESI†) with the accompanying Raman Toolkit web interface to build SOM models using training data and perform predictions against test data. SKiNET models were optimised by performing 10-fold cross validation on the training data, and tuning the number of neurons, initial learning rate and number of training steps. 20% of the collected spectra per tissue type per disease class were randomly selected to be used for testing data whilst the remaining 80% were used for training. The optimised model was used to classify the previously unused test data. Classification using the test data was repeated ten times from separate SOM initialisations.

## Results and discussion

### Tissue type classification and morphology

Tissue types were classified according to the convention of Plymale and Wylie,^36^ where the primary vein, or mid-vein, is the one extending directly from the base of the leaf into the blade, the secondary veins, or lateral veins, are veins branching directly from the mid-vein and the veins branching directly from the lateral veins are described as the tertiary veins, collectively referred to as venules, based on the precedence by Yates and Duncan^37^ and Silva *et al.*^38^ The remaining mesophyll tissue surrounding the veins is classified as “non-vein” to distinguish from the venous tissue.

Plant tissue is primarily composed of three classes of cells including the parenchyma, sclerenchyma and collenchyma at varying distributions. NV tissue contains a blend of collenchyma, parenchyma and chlorenchyma (chloroplast-containing parenchyma).^39^ Moving from Ven to LV to MV tissue, there is a decreasing amount of collenchyma and an increasing amount of parenchyma.^36,40^ Furthermore, venous tissue contains the xylem and phloem, vessels to transport water into the leaf and photosynthates out of the leaf, respectively, surrounded by sclerenchyma giving a “ribbed” appearance to venous tissue.^41^ The combination of these varying degrees of the three main cell types, results in the differences on the microscopic level (Fig. 1).

### Spectral fingerprints and differences of HOL, NMCOL, MCOL

**Fig. 2:**
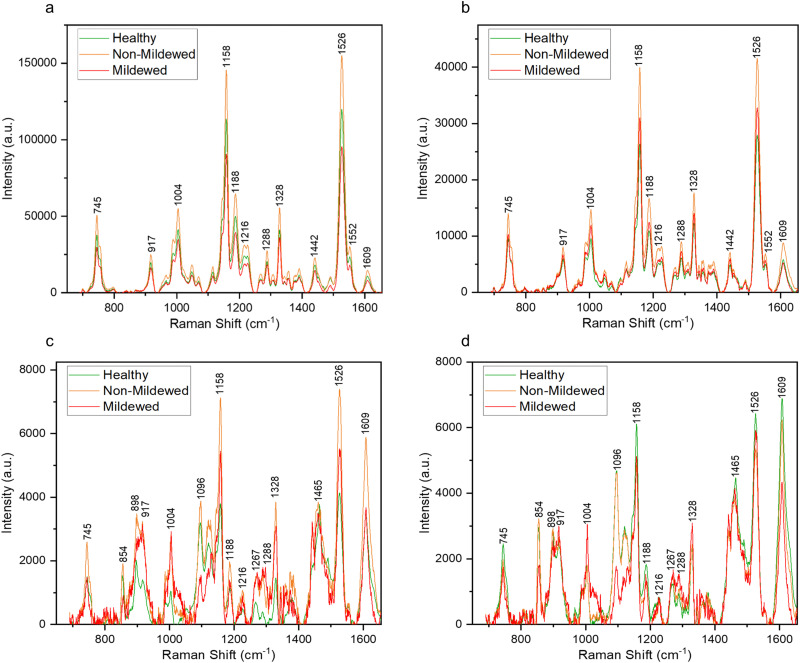
Average Raman spectra of the representative shift of the characteristic peaks present obtained from areas of (a) non-vein, (b) venule, (c) lateral vein and (d) mid-vein tissue types on the *Q. robur* leaf, which were either from a healthy leaf or an infected leaf, distinguishing between mildew-covered and non-mildew covered areas of infected leaves.

Raman spectra collected from the oak leaf samples included peaks associated with common plant biomolecules such as structural biomolecules, *i.e.*, cellulose and lignin, and functional biomolecules, *i.e.*, chlorophyll and carotenoids (Table 4). Biomolecular changes caused by the interactions between oak leaves and the oak PM were clearly identifiable in these spectra (Fig. 2). Raman spectra with standard deviation shading are shown in Fig. S2 (ESI†).

### Non-vein and venule tissue types

Structural peaks identified in NV and Ven tissue types (Fig. 2a and b) are attributed to: pectin at 745 cm^−1^,^42^ cellulose and lignin at 917 cm^−1^,^43^ lignin at 1188 cm^−1^,^44^ lignin and xylan at 1216 cm^−1^,^45^ cellulose and lignin^19,43^ with a weak contribution of pectin^42^ at 1328 cm^−1^ and lignin^46^ with a weak contribution of pectin^42^ at 1609 cm^−1^. Functional peaks determined were carotenoids at 1004, 1158 and 1526 cm^−1^ ^47^ and chlorophylls at 1188 and 1552 cm^−1^.^48^ Additional bands identified included two aliphatic peaks at 1288 and 1442 cm^−1^.^49^

For NV tissue, a change in global intensity across the whole range of wavenumbers from HOL and NMCOL to MCOL samples was observed with significant decreases in range from HOL and NMCOL to MCOL tissue and a significant increase in range from HOL to NMCOL tissue (Table 2 and Fig. S3, ESI†). This indicates chlorosis, the loss of chlorophyll, occurring in mildew-covered tissue as chlorophyll has a residual fluorescence at 830 nm, artificially increasing the Raman profile.^23,24^ Chlorosis was confirmed using UV-Vis spectrophotometry (Table 3 and Fig. S4, ESI†) with significant decreases in concentration noted from NV and Ven tissues of HOL and NMCOL tissues relative to those of MCOL tissue only for chlorophyll *b* and from HOL to MCOL tissue for chlorophyll *a* + *b*. Chlorophyll *a* and chlorophyll *b* differ by only one side chain, methyl compared to formyl, respectively,^50^ however chlorophyll *a* is the main photosynthetic agent whereby, chlorophyll *b* is an accessory photosynthetic agent similar to the carotenoids.^51^ Hence, a significant reduction in chlorophyll *b* concentration indicates that the *E. alphitoides* pathogen is reducing the photosynthetic efficiency of oak PM affected leaves whilst still retaining the major photosynthetic component, chlorophyll *a*, for absorption of photosynthates for growth. These findings are in correspondence with the previous study by Skwarek-Fadecka *et al.*,^14^ which ascertained chlorophyll levels in oak PM affected leaves. It should be noted that there are currently conflicting opinions about the diagnostic value of this global decrease and by extension chlorosis, through infection,^23,24^ due to the large variability in chlorophyll concentration within the leaf caused by factors such as leaf age and the location where the leaf is being grown. No further spectral differences were detected between HOL, NMCOL or MCOL samples and no mildew-specific peaks were detected on HOL, NMCOL or MCOL spectra for both NV and Ven sample spectra.

**Table 2:** Average ranges of Raman intensities and coefficients of variation for non-vein tissue from *Q. robur* leaves for each disease class. 1, 2 and 3 superscripts denote significantly different pairs of ranges (*p* < 0.005) identified using a Mann–Whitney *U* test

Disease class	Average range of Raman intensities (a.u.)	Coefficient of variation (%)
Healthy	121 989 ± 54 587^1,3^	44.75
Non-mildew covered	156 921 ± 41 864^1,2^	26.68
Mildew covered	97 381 ± 40 105^2,3^	41.18

**Table 3:** Concentrations of chlorophyll *a*, chlorophyll *b* and total chlorophyll (*a* + *b*) for *Q. robur* leaves. α, β and γ superscripts denote significantly different pairs of concentrations (*p* < 0.05) identified using a Mann–Whitney *U* test

Disease class	Chlorophyll concentration (μg mL^−1^)		
	*a*	*b*	*a* + *b*
Healthy	32.29 ± 4.64	15.36 ± 3.16^α^	47.6 ± 5.41^γ^
Non-mildew covered	30.83 ± 3.46	17.31 ± 4.89^β^	48.15 ± 7.45
Mildew covered	27.45 ± 2.31	13.03 ± 2.39^α,β^	40.48 ± 4.24^γ^

**Table 4:** Identified characteristic peaks with the corresponding assignments of *Q. robur* leaves

Raman shift (cm^−1^)	Tentative assignment	Bond vibration	Tissue type	Ref.
745	Pectins	*γ*(C–OH)_COOH_	All	42
854	Pectins	C–C–O–C–O skeleton	LV, MV	42
898	Cellulose	Not identified	LV, MV	45
	Pectins	*δ*(C–C–H) + *δ*(C–O–H)		42
	Xylan	*δ*(CH)_aromatic_		52
917	Cellulose	Not identified	All	43
	Lignin	*ν*(C–O–C)_symm_		
1004	Carotenoids	*ρ*(CH_3_)_polyene_	All	47
1096	Cellulose	Not identified	LV, MV	53
1120	Cellulose	Not identified	LV, MV	45
	Xylan	*δ*(C–O–C) + *δ*(C–C)		45 and 52
1127	Xylan	*δ*(C–O–C) + *δ*(C–C)	LV, MV	45 and 52
1158	Carotenoids	ν(C–C)	All	47
1188	Chlorophyll lignin	*ν*(C–N)	All	48
		*ν*(C–O–H)_aromatic_		44
1216	Lignin, xylan	Not identified	All	45
1267	Glucomannan	Not identified	LV, MV	45
1288	Aliphatics	*δ*(CH_2_) + *δ*(CH_3_)	All	49
1328	Cellulose	*δ*(CH_2_)	All	19 and 43
	Lignin	*δ*(CH_2_)		19 and 43
	Pectins	*δ*(CH)		42
1442	Aliphatics	*δ*(CH_3_)_asymm_	NV, Ven	49
1465	Aliphatics	*δ*(CH_2_) + *δ*(CH_3_)	LV, MV	49
1526	Carotenoids	*ν*(C=C)	All	47
1552	Chlorophyll	*ν*(C–C)_pyrrole_	NV, Ven	48
1609	Lignin	*ν*(C–C)_aromatic_	All	46
	Pectins	*ν*(COO^−^)_asymm_		42

The reproducibility of the RS method was established on the NV tissue by calculating the 1526/1158 ratio from measurements taken from HOL, NMCOL and MCOL *Q. robur* samples and comparing between different leaves collected on different days (*n* = 16). The reproducibility constant (Fig. S5 and Table S5.1, ESI†) was found to be 12.35%, 7.54% and 8.83% for HOL, NMCOL and MCOL samples, respectively, indicating low variability in the measurements between different leaves and samples.

### Lateral vein and mid-vein tissue types

**Fig. 3:**
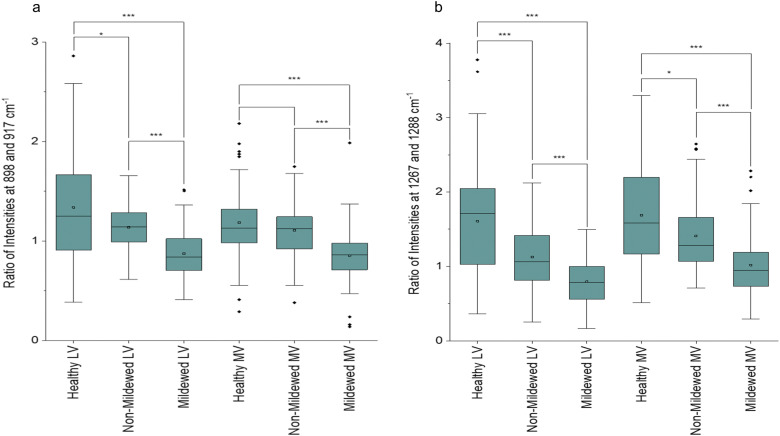
Box and whisker plots of the ratio of intensities of (a) 898 and 917 cm^−1^ and (b) 1267 and 1288 cm^−1^ peak pairs for healthy, non-mildew covered and mildew-covered lateral vein and mid-vein *Q. robur* leaf tissue samples. Brackets represent significantly different pairs of ratios compared *via* a Mann–Whitney *U* test with p* < 0.05, p*** < 0.005.

In addition to the NV and Ven identified peaks of importance, in LV and MV spectra (Fig. 2d) new structural peaks of: pectin at 854 cm^−1^;^42^ cellulose, pectin and xylan at 898 cm^−1^;^42,45,52^ cellulose at 1096 cm^−1^;^53^ cellulose and xylan at 1120 and 1127 cm^−1^ ^45,52^ and glucomannan at 1267 cm^−1^ ^45^ were detected. There is a further new aliphatic peak at 1465 cm^−1^.^49^ For these tissues, chlorosis was also noted to a lesser degree, due to the lower amount of chlorophyll present in LV and MV relative to NV and Ven tissues. Unlike NV and Ven tissues, there were spectral differences between HOL and NMCOL, HOL and MCOL and NMCOL and MCOL samples. These differences were mainly attributed to the following pairs of bands, the 898 and 917 cm^−1^, the 1120 and 1127 cm^−1^ and the 1267 and 1288 cm^−1^, with the 898/917 and 1267/1288 pairs representing differences in intensity and the 1120/1127 pair representing a difference in the wavenumber. The intensity differences are summarised in Table 5 and analysed in Fig. 3.

**Table 5:** Intensity ratios of 898/917 cm^−1^ and 1267/1288 cm^−1^ peak pairs for *Q. robur* lateral vein and mid-vein tissues

Disease class	Ratio of 898/917		Ratio of 1267/1288	
	Lateral vein	Mid-vein	Lateral vein	Mid-vein
Healthy	1.34 ± 0.53	1.19 ± 0.35	1.61 ± 0.70	1.69 ± 0.71
Non-mildew covered	1.14 ± 0.23	1.11 ± 0.29	1.13 ± 0.43	1.41 ± 0.45
Mildew covered	0.87 ± 0.24	0.85 ± 0.27	0.80 ± 0.30	1.02 ± 0.41

The average HOL and NMCOL intensity ratios, yield a value of >1, the LV MCOL intensity ratio yields a value <1 and the MV MCOL sample ratio a value of ≤1 for the 898/917 ratio and ≥1 for the 1267/1288 ratio. Mann–Whitney *U* tests identified that for the 898/917 ratio, there are significant decreases from HOL to NMCOL (*p* < 0.05), NMCOL to MCOL (*p* < 0.005) and HOL to MCOL LV samples (*p* < 0.005) and significant decreases from NMCOL to MCOL (*p* < 0.005) and HOL to MCOL MV (*p* < 0.005). For the 1267/1288 ratio, there are significant decreases from HOL to NMCOL, NMCOL to MCOL and HOL to MCOL LV samples (*p* < 0.005) and from HOL to NMCOL (*p* < 0.05), NMCOL to MCOL (*p* < 0.005) and HOL to MCOL MV (*p* < 0.005). Thus, indicating biomolecular similarity between HOL and NMCOL samples for MV tissue and dissimilarity for LV tissue and between these and the MCOL samples on both lateral vein and mid-vein tissues. The dissimilarity between the 898/917 and 1267/1288 ratios for HOL and NMCOL LV samples is evident through the increased intensities in all four of these peaks (Fig. 2c).

The NMCOL spectra could either be locally healthy or locally infected, yet asymptomatic, due to the proximity of the mildew to the non-mildew covered sampling points. This dissimilarity is most likely due to a plant defence reaction, the hypersensitive response, preventing global infection of the leaf, which has been observed in other powdery mildew-plant interactions in multiple pairs of species.^54,55^ This response is a local senescence reaction by the plant cells to restrict fungal growth within the plant tissue.

This response also triggers a regulation of the defence response in distant tissue as well as locally in the infected tissue and thus, the proximity to mildew-covered tissue would present as locally healthy due to the hypersensitive response,^56^ explaining the changes seen in intensity ratios in this study.

The 1288 cm^−1^ aliphatic peak also differed in MCOL samples, associated with the highly common CH_2_ and CH_3_ groups. This band is associated with the cuticular and epicuticular layers of the leaf, which can change through plant pathogen infection.^57^ Further detected changes included the 1004, 1158 and 1526 cm^−1^ carotenoid peak intensities. However, 1158 and 1526 cm^−1^ peaks did not decrease as much as previously observed,^20,23,57,58^ where decreases in carotenoid were determined to be either the defence-instigated repurposing of carotenoids into apocarotenoids or pathogen-caused degradation.

The increase of the 1004 cm^−1^ peak corresponds with the observations of Rys *et al.*^59^ who noted that the foliar pathogen, *Obuda pepper virus*, caused a significant increase of the 1005, 1156 and 1525 cm^−1^ carotenoid peaks, likely due to dehydration of the leaf caused by the hypersensitive response. A similar effect was observed in this study, though further work to determine the water content of the leaf through infection will be necessary to confirm this hypothesis. A decrease of the 1096 and 1120 cm^−1^ cellulose peaks was observed in LV and MV tissue types whilst the 898 cm^−1^ peak only decreased in MV tissue. The hydrolysis of cellulose by plant pathogens is a common occurrence^23,57^ as well as the hydrolysis of pectins, which was noticed by the decrease of intensity of the 854 cm^−1^ peak, indicating a reduction of the C–C–O–C–O skeletons of pectin molecules. Pectins are hydrolysed by *E. alphitoides* to remove ester linkages hence enabling further fungal colonisation of the plant tissue.^60^

Similar to the degradation of cellulose, degradation of hemicelluloses, such as xylan or glucomannan, most probably by pathogen-induced hydrolysis, is common in plant pathogen–plant host interactions.^23,57^ However, an increase of the intensity of xylan peaks, especially at 1127 cm^−1^, and the 1267 cm^−1^ glucomannan peak in both tissue types was observed in our MCOL samples. This discrepancy between cellulose and hemicellulose changes is not currently understood and further research is needed to further explain these interactions.

An increase of the 917 and 1328 cm^−1^ lignin peaks and the appearance of a 1270 cm^−1^ lignin peak across both tissue types was also observed. This is most likely due to the lignification of cell walls, a defence response used by plants triggered by the presence of an invading pathogen.^61,62^ Lignification is often a compensatory mechanism given the increased susceptibility of the plant cells by the loss of cellulose and pectin.^60^

Finally, numerous new minor peaks were observed in both LV and MV tissues for the MCOL samples with several larger peak areas. These are likely due to an increased metabolite activity in the leaf samples during infection as a defence response. Metabolite activity can bring numerous new Raman active molecules to the LV and MV regions resulting in many new bond types and thus, new peaks appearing throughout locally infected samples. This could also be a reason for the increased presence of CH_2_ and CH_3_ groups. Also, similarly to NV and Ven tissue spectra, there were no mildew-specific peaks detected on HOL, NMCOL or MCOL spectra for either LV or MV tissue.

### Spectral classifications

**Fig. 4:**
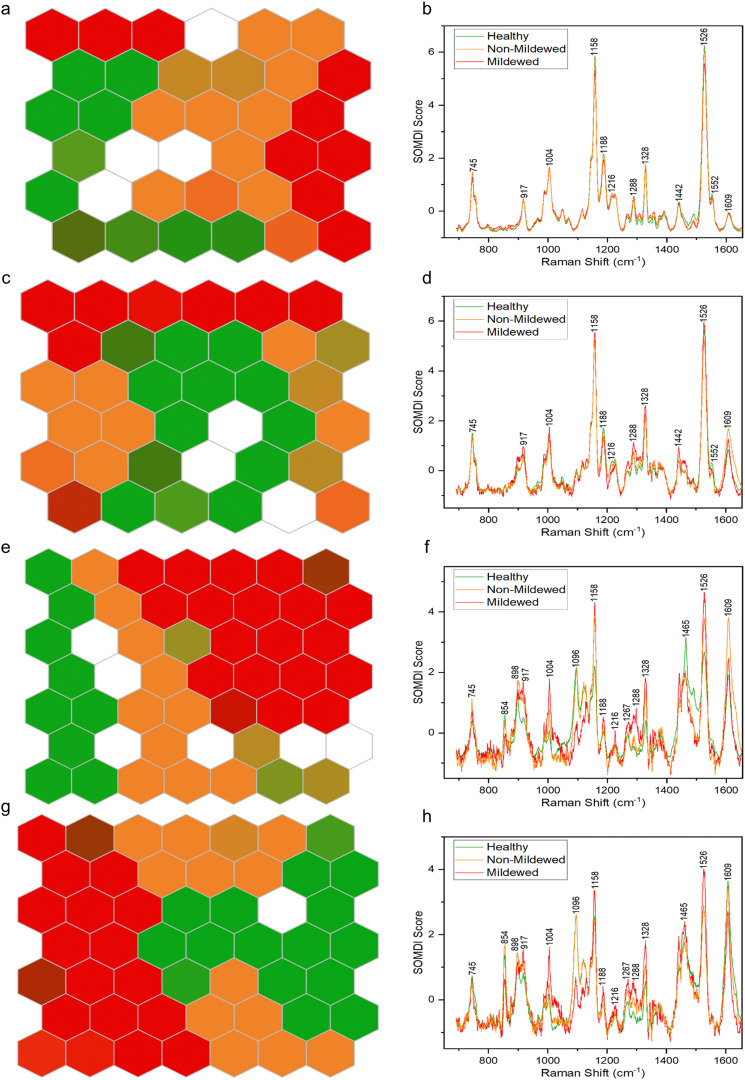
Clustering of Raman spectra for (a) non-vein, (c) venule, (e) lateral vein and (g) mid-vein tissues of *Q. robur* leaves for healthy tissue (green), mildew-covered tissue (red) and non-mildew covered tissue (orange) *via* SOMs. The corresponding SOMDI extracted features highlight the most important Raman peaks for different disease classifications in (b) non-vein, (d) venule, (f) lateral vein and (h) mid-vein tissues of a *Q. robur* leaf.

By using SKiNET the separability of the tissue type spectral classes can be visualised as an array of hexagons showing inter- and intra-class mixing. Fig. 4 shows differing degrees of separability between the four tissue types, arranged as 6 × 6 (Fig. 4a and c) or 7 × 7 (Fig. 4e and g) SOMs. White hexagons (8% of data in total) represent the neurons in which there was no majority class from the training data to activate them.

The NV tissue 6 × 6 SOM (Fig. 4a) appears to be poorly separated, with areas of connecting same-colour neurons and mixed colour hexagons, signifying a large degree of spectral similarity from the three disease state classes. This concurs with the spectral analysis, showing no wavenumber or intensity change between these spectra. The MCOL assigned hexagons on the other hand, are represented as two distinct areas, indicating an external variable creating two classes of MCOL data. The data classification accuracy in this case was found to be 69.6 ± 3.17%. The importance of the 1158 and 1526 cm^−1^ carotenoid peaks to the separation of the data (Fig. 4b) indicates that the differences between these disease class spectra is likely due to chlorosis. These peaks decrease the most between globally or locally healthy samples and mildew-covered samples.

The Ven tissue 6 × 6 SOM (Fig. 4c) contains a large adjoining block of green hexagons, continuously connecting orange and red hexagons, mixed-colour hexagons and a degree of dispersion, particularly for the orange hexagons. This indicates separation between HOL and infected class spectra, with a degree of similarity between MCOL and NMCOL groups. Further variability in the NMCOL samples is established given the degree of dispersion of hexagons belonging to that class. The classification accuracy was found at 73.5 ± 2.99%, with the 1158 and 1526 cm^−1^ carotenoid peaks being the most significant for separating the classes (Fig. 4d). Like the NV tissue, these peaks exhibited the biggest decrease, indicating chlorosis as the most likely cause for the differentiation.

The LV tissue 7 × 7 SOM (Fig. 4e) exhibits a fully contiguous block of red hexagons and mostly adjoining blocks of green and orange hexagons, with several mixed-colour hexagons. This indicates a clear separation between the three diseases classes with a little spectral similarity between healthy and non-mildew covered sample spectra. The accuracy in this case was established to be 82.1 ± 1.91%. The most important bands responsible for the class separation include the 1158 and 1526 cm^−1^ carotenoid and the 1609 cm^−1^ lignin peaks (Fig. 4f), due to the changes observed in these regions from chlorosis and lignification of cell walls.

The MV tissue, 7 × 7 SOM (Fig. 4g) contains adjacent blocks of green and red hexagons with a degree of spread in orange and mixed-colour hexagons with a classification accuracy of 85.6 ± 3.31%. This indicates a good separability between disease classes and a small degree of sample variation between the non-mildew covered tissues. The most important SOMDI identified peaks, like the LV tissue, are the 1158 and 1526 cm^−1^ carotenoid and the 1609 cm^−1^ lignin peaks (Fig. 4h), also associated with chlorosis and the lignification of cell walls.

### Wetting and adhesion properties

**Fig. 5:**
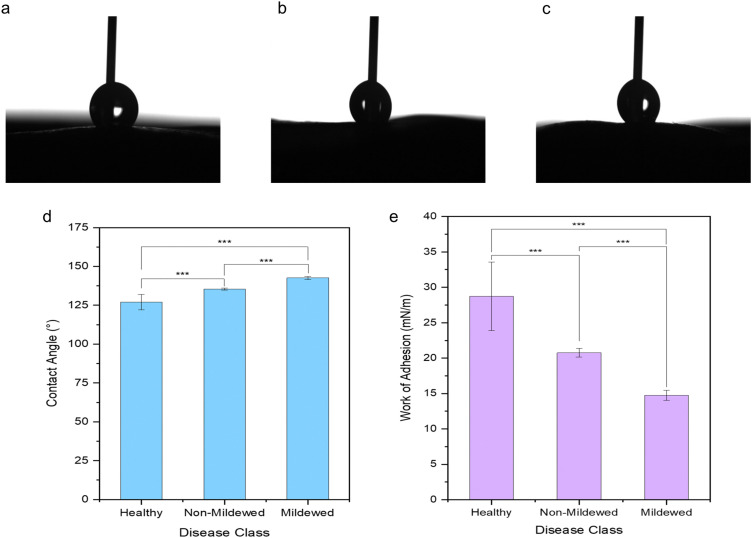
Optical images of static contact angle measurement water drops on (a) healthy, (b) non-mildew covered and (c) mildew-covered non-vein and venule tissues of *Q. robur* leaves with the corresponding (d) contact angle measurements and the (e) calculated work of adhesion, highlighting the statistically significantly differences identified *via* Mann–Whitney *U* tests (p*** < 0.005).

It is evident that the wetting behaviour is considerably altered by the surface structure and dimensions due to the mildew presence in comparison to the healthy controls of the same leaf (Fig. 5) with water drops formed on the leaves being visually distinct (Fig. 5a–c) as the hydrophobicity changes. Lower contact angles of 127.10 ± 4.89° are measured on healthy leaves (non-wettable), increasing to 135.37 ± 0.69° on non-mildew covered leaves (highly non-wettable) and onto 142.67 ± 0.95° on mildew-covered leaves (highly non-wettable) (Fig. 5d).

The work of adhesion of these leaves was found to be 28.73 ± 4.84 mN m^−1^ on healthy leaves, decreasing to 20.76 ± 0.60 mN m^−1^ on non-mildew covered leaves and to 14.75 ± 0.72 mN m^−1^ on mildew-covered leaves. These findings are attributed to the presence of mildew on the oak leaves, known to increase the hydrophobicity and in turn, decrease the ability of the leaves to retain water drops on their surfaces^26,27^ with the observed increase most likely to be due to the presence of the hierarchical structure of the mildew on the leaf^27^ (Fig. 1b, d and f). However, this does not explain the significant increase of hydrophobicity measured on non-mildew covered areas of infected leaves. Although the presence of the hypersensitivity response on LV and MV tissues affects distant tissue as well as tissue local to the site of infection, it is currently unknown if these hypersensitivity response-related biomolecular changes alter the surface chemistry or ultrastructure of the cuticular and epicuticular waxes on the leaves, which have been identified as key markers of wettability changes.^27^ The changes determined *via* Raman spectra of LV and MV tissues (Fig. 2c and d) indicate changes in the aliphatic bonds, which can be attributed to epicuticular waxes^49^ however, these are not seen for NV and Ven tissues (Fig. 2a and b). The non-mildew covered tissue (Fig. 5d and e) indicates that there may be undetected changes to these epicuticular wax-related peaks in the Raman spectra for NV and Ven tissues, providing further insights into the oak PM-oak leaf interaction. The statistically significant differences determined between the measured contact angle and work of adhesions of HOL, NMCOL and MCOL samples further correspond with SOM clustering and overall SKiNET classification results (Fig. 4a, c, e and g).

Overall, from this investigation *via* non-invasive Raman spectroscopy to determine the unique chemical fingerprints defined by the underpinning molecular constituents, and in combination with SKiNET AI, enabling the rapid and accurate identification and study of the underpinning mechanism and changes, it has been established that non-mildew covered areas of *E. alphitoides* infected *Q. robur* leaves are locally healthy, likely due to the hypersensitive response. This has been determined from a combination of the biomolecular similarities between healthy and non-mildew covered oak leaf tissues observed through the lack of significant changes in chlorophyll concentrations in the non-vein and venule tissues and an increase to the 1004 cm^−1^ peak in the lateral vein and mid-vein tissues, indicating dehydration of the leaf tissue, both common signs of the hypersensitive response.

Non-vein and venule tissue-specific biomolecular changes were further noted, due to the combined presence of *E. alphitoides* mildew and the hypersensitive response, predominantly leading to chlorosis of infected leaves. Analysing the non-vein tissue Raman spectra, revealed a decrease in global intensity across all wavenumbers as the primary change moving from healthy and non-mildew covered to mildew-covered leaf tissue. This has been linked to chlorosis and confirmed using UV-Vis spectrophotometric measurements of non-vein and venule tissues from these leaves, specifically for the chlorosis of chlorophyll *b* in mildew-covered tissue, reducing the photosynthetic efficiency of infected leaf tissue. This global decrease of intensities was not observed in the venule tissue Raman spectra, likely caused by the differing concentrations of chlorophyll in the samples measured due to leaf age and growing conditions, amongst other external factors. For non-vein and venule tissue, the non-mildew covered tissue spectra were higher in intensity overall compared to the healthy tissue. Given that non-mildew covered tissue has been shown to be locally healthy, this increase is most likely due to leaf age affecting the chlorophyll concentration within the leaf samples and thus, yielding in the highest intensity arising from the non-mildew covered tissue spectra. Additionally, high coefficients of variance were noted for healthy and mildew-covered samples at 44.75% and 41.18% respectively, most likely due to the high degree of natural variation combined with leaf ages affecting chlorophyll concentrations and thus ultimately, Raman signal intensities. No biopolymer-specific changes were identified *via* the spectral signatures of non-vein or venule tissues, indicating no material change to the chlorenchyma, collenchyma or parenchyma cells in these tissues. Given these cell types contain lignified primary cell walls,^63,64^ no lignin-related changes were identified *via* Raman spectra, indicating no changes to the lignified primary cell walls present in the cells of these tissues. No mildew-specific peaks were identified in spectra from non-vein or venule tissues.

Lateral vein and mid-vein tissue resulted in more complex biomolecular changes throughout infection. The changes to the 1288 cm^−1^ aliphatic peak in mildew-covered tissue indicate changes in the cuticular and epicuticular layers of the leaf tissue through infection, likely in response to the presence of the pathogen. Changes to the 1004, 1158 and 1526 cm^−1^ carotenoid peaks indicate either defence-instigated repurposing or pathogen-instigated degradation of carotenoids, ultimately affecting photosynthetic efficiency. The 1096 and 1120 cm^−1^ cellulose peaks decreased in both tissues whilst the 898 cm^−1^ cellulose, pectin and xylan peak only decreased in mid-vein tissue. This indicates the hydrolysis of cellulose and pectin by the *E. alphitoides* fungus, to allow further fungal colonisation of the plant tissue. The 1127 cm^−1^ xylan and the 1267 cm^−1^ glucomannan peaks appear to increase in intensity, which contradicts with the cellulosic degradation as typically both hemicellulose and cellulose degrade at similar rates due to pathogen-produced enzymes. An increase of the 917 and 1328 cm^−1^ lignin peaks with a new 1270 cm^−1^ lignin peak were also detected, indicating defence-instigated lignification of cell walls in response to the pathogen. Finally, numerous new peaks were present in both spectra for the mildew-covered tissue, likely due to increased metabolite activity in the venous tissue in response to infection. Considering these tissue types are low in collenchyma and high in parenchyma and sclerenchyma, whereby the parenchyma contains lignified primary cell walls and sclerenchyma contains lignified secondary cell walls,^63,64^ detected differences indicate changes to the parenchyma, which is high in cellulose and pectin,^40^ given the high degree of change to these biopolymers. Therefore, it is highly likely that the defence-instigated lignification of cell walls occurs within the primary cell walls of the parenchyma. Similarly, like non-vein and venule tissues, the spectrum from non-mildew covered lateral vein tissue was higher in intensity than the healthy lateral vein tissue. Since the non-mildew covered tissue is locally healthy, this is also due to the leaf age-related changes to chlorophyll concentrations leading to a heightened response compared to healthy tissue and thus, the highest spectral intensities. Finally, in correspondence with the non-vein and venule tissue spectra, no mildew-specific peaks were identified on lateral vein and mid-vein spectra.

Furthermore, the presence of *E. alphitoides* mildew on the oak leaves has been found to increase the hydrophobicity of these leaves and thus, decrease their ability to retain water drops on their surfaces. Similar changes were noted on non-mildew covered areas of *E. alphitoides* infected leaves albeit to a lesser degree. These identified changes in wetting states have been attributed to the changing competition of forces acting on the water-leaf interface, caused by changes to foliar surface roughness by the local or global presence of filamentous fungal structures and impregnation of the hierarchical fungal structures by water or air. The presence of micro- and nano-epicuticular wax ultrastructures in healthy oak leaves results in apolar foliar surfaces with high contact angles due to low adhesion on the liquid-leaf interface, which appears to be exacerbated by additional micro- and nano-filamentous fungal structures from *E. alphitoides* present on the mildew-covered samples. There is a lack of micro- and nano-filamentous fungal structures from *E. alphitoides* on non-mildew covered samples, however, the enhanced apolarity of these samples compared to healthy leaves was identified, most likely caused by an outcome of the hypersensitive response, laying the groundwork for further needed work to understand this process.

## Conclusions

Herein, we have investigated foliar interactions on a tissue-by-tissue basis between a forest foliar disease, oak powdery mildew, and English oak leaves. Raman spectroscopy combined with an advanced artificial neural network algorithm and interfacial wetting property determination has been applied to investigate whether the identified biomolecular changes of *Quercus robur* leaves by *Erysiphe alphitoides* reflect and distinguish healthy leaves from infected leaf states.

Classification of the Raman spectroscopic profiles of these disease classes *via* the SKiNET algorithm provided identification accuracies of 69.6% for non-vein, 73.5% for venule, 82.1% for lateral vein and 85.6% for mid-vein tissues. For lateral vein and mid-vein tissue, the intensity of the band ratios at 898 and 917 cm^−1^ as well as at 1267 and 1288 cm^−1^, attributed to various plant biopolymer and cuticular wax components, have been found to be the key-differentiating dominant peaks, accurately distinguishing between disease classes. Changes to interfacial wetting properties between healthy, non-mildew and mildew-covered areas of *Quercus robur* leaves further echo these differences established *via* the SKiNET algorithm.

Thus, Raman spectroscopy has been successfully demonstrated as a specific and non-invasive technique for the detection and multiplexed profiling of foliar forest pathogen–host tissue interactions. Rapid differentiation of biomolecular compositions has been enabled from the acquired spectral profiles resulting in the identification of changing biomolecules of interest. Probing these interactions between the foliar forest pathogen, *Erysiphe alphitoides* and *Quercus robur* host leaves, whilst simultaneously cataloguing biomolecular-level interactions, lays the platform for exploring interactions in other tree-forest pathogen systems and between biological soft matter by extension. This versatile approach, alongside automating the classification of Raman spectra and assignment to a particular leaf and disease state, would form the further important step for the translation of Raman-based foliar forest disease-diagnostic techniques to real world, point-of-need applications for rapid diagnoses of forest pathogens and potentially lay the groundwork for targeted treatments of these forest pathogens.

## Conflicts of interest

There are no conflicts to declare.

## Supplementary Material

SM-020-D3SM01392H-s001
