# Supplementary material for: Vibrational spectroscopic profiling of biomolecular interactions between oak powdery mildew and oak leaves
Source: Soft Matter. 2024 Jan 3;20(5):959–70. doi: 10.1039/d3sm01392h (PMC10828924; doi:10.1039/d3sm01392h)
Supplement: SM-020-D3SM01392H-s001 [file SM-020-D3SM01392H-s001.pdf]

Electronic Supplementary Material (ESI) for Soft Matter.
This journal is © The Royal Society of Chemistry 2024

# - Supplementary Information -

Vibrational Spectroscopic Profiling of Biomolecular Interactions Between Oak Powdery Mildew and Oak Leaves

Kieran R. Clark and Pola Goldberg Oppenheimer

S1. Self-Optimising Kohonen Index Network (SKiNET) Algorithm:

The developed artificial neural network (ANN) algorithm, the self-optimising Kohonen index network (SKiNET) classifies presented data using self-organising maps (SOMs) and provides classification support through self-organising map discriminant indices (SOMDIs), acting as an inherent decision support tool.

Overview

The SKiNET algorithm is a framework which provides dimensionality reduction, feature extraction and multiclass classification, whereby the algorithm performs and provides visual separation to highlight underlying biomolecular differences between Raman spectra of different classes. This results in accurate classification of the inherently information-rich data that are Raman spectra, which are usually difficult to interpret for large sample numbers with a high degree of specificity.

Unlike conventional SOM algorithms, SKiNET provides supervised learning combined with 10-fold cross validation, which helps to provide consistently accurate classification of Raman spectra into different disease classes whilst simultaneously providing SOMDIs which help the user identify peaks which are class-defining and thus are the most important for classification and ultimately, separation between disease classes or lack thereof.

Details of SKiNET

SOMs are inspired by the visual cortex of the brain and are designed to learn from data autonomously. ANNs operate by iteratively adjusting weights and biases of neurons to achieve a desired objective through epochs, this process is known as training the ANN algorithm. In the case of SOMs, this training process is designed to allow inputs with similar characteristics to activate neighbouring neurons.

In this study, the inputs for the SKiNET algorithm are normalised Raman spectra. Neurons in the SKiNET algorithm possess weight vectors with a dimensionality equal to the number of variables present in the Raman spectrum. Throughout the training process, these weight vectors are varied by examining the differences between neuron weights and class weight vectors, until they closely match the input training spectra such that each neuron will only activate on a spectrum from a single class. This is shown visually as a cluster which can be identified as from a given sample classification.

This training process enables the detection of which class-defining characteristics activate specific neurons by inspection of the weights across all neurons and isolating those which belong to a specific class. Upon inputting unseen testing data into the algorithm, if class-defining characteristics are present, these will activate those neurons which match to those characteristics thus, providing a sorting of that data to a class / group / state / disease classification.

For the SKiNET algorithm, the training parameters are the grid size, learning rate and number of epochs the network is trained over. The outcome of this SOM process is a visual representation of the separability of the testing spectra into classes, ultimately showing biomolecular similarities or dissimilarities between the spectra.

These class-defining characteristics are shown as peaks on a SOMDI plot against wavenumber (in cm^{-1}) and relate to those peaks in the testing or training spectra, which contribute most to the activation of class label-specific neurons and ultimately, the SOM clustering.

Specifically, in the ***'Vibrational Spectroscopic Profiling of Biomolecular Interactions Between Oak Powdery Mildew and Oak Leaves'*** study, the process of applying SKiNET to achieve hybrid Raman spectroscopy was used to investigate potential biomolecular similarity or dissimilarity between healthy, non-mildew covered and mildew-covered tissues of *Erysiphe alphitoides* infected *Quercus robur* leaves, whilst *simultaneously* providing which characteristic peaks were important / dominant in achieving this separation, if present.

SKiNET enabled this by providing SOMs comprised of a hexagonal grid, where each hexagon represents a given neuron in the model. Each neuron was coloured according to the disease classification they activate, given by the class-defining characteristics, provided by the inputted training spectra. In this study, the training spectra were composed of 80% of the total 70-75 spectra from tissues which were healthy, non-mildew covered or mildew-covered assigned using a visual and microscopic survey of the surface of the leaf for the presence of mildew or the lack thereof. White neurons in the hexagonal grid express neurons that did not have a majority class or did not activate with any of the class-defining characteristics. Mixed colour neurons in the hexagonal grid express neurons which had multiple class-defining characteristics so could not be given a single colour for a single disease classification.

This results in three options of hexagons present in SOM, *i.e.,* a coloured hexagon belonging to a single class classification, a mixed colour hexagon belonging to one or more class classification or a white hexagon belonging to no class classification. Thus, by examining contiguous or connected blocks of hexagons of single colours, along with the number of white hexagons and the number and condition of mixed colour hexagons, if they exist, it is possible to rapidly ascertain the degree of biomolecular similarity or dissimilarity between spectra of different disease / class classifications.

Finally, the inherent SOMDI component of the SKiNET plotted as index *versus* the wavenumber (cm^{-1}) allows the assessment of peaks of importance for the separability of spectra from different classifications, with a higher SOMDI value attributed to a higher importance which has led to the class separation and classification in SOM.

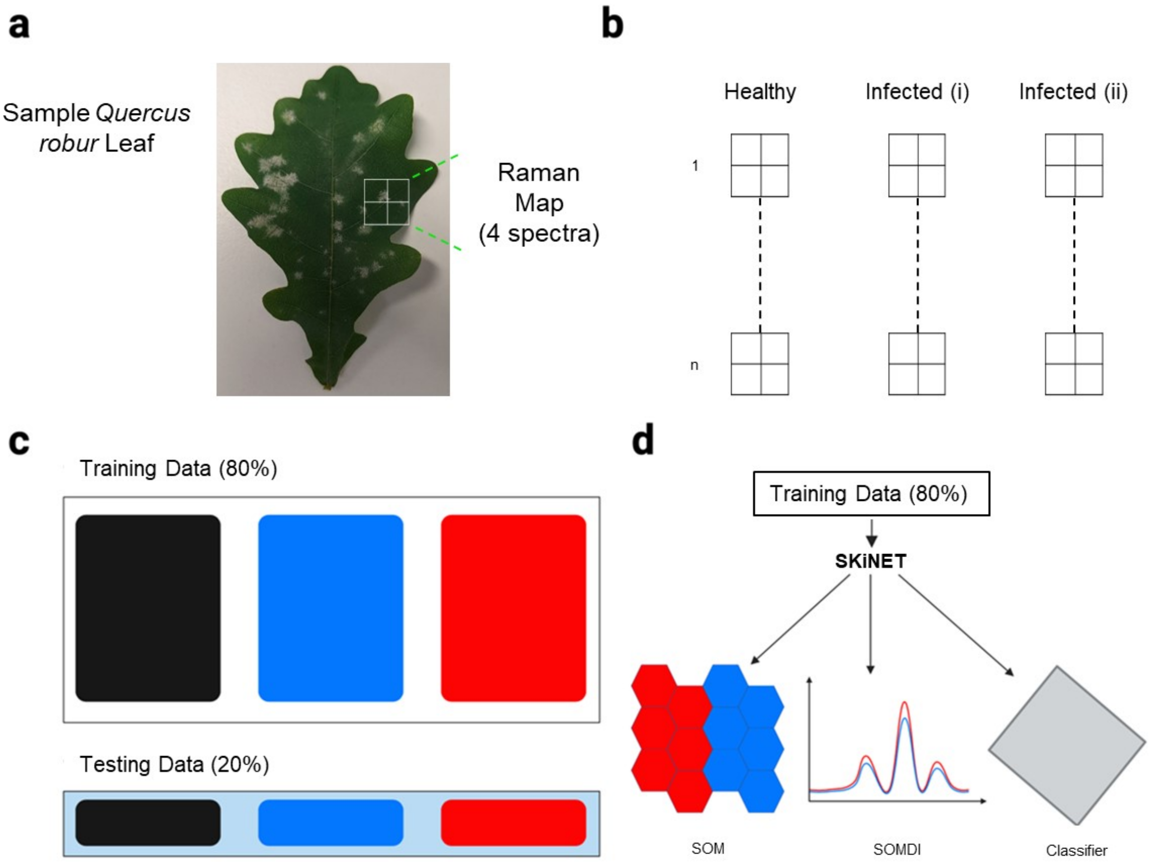

Figure S1. Illustration of a workflow for data analysis pipeline *via* SKiNET. Raman spectra measured from non-vein, venule, lateral vein or mid-vein tissue of a *Quercus robur* leaf **(a).** These spectra are grouped according to disease

class **(b)**, either healthy, non-mildew covered (Infected (i)) or mildew covered (Infected (ii)). This data is then split 80/20 randomly with the 20% reserved as unseen spectra for testing **(c)**. The remaining 80% is then inputted into SKiNET, which directly provides dimensionality reduction (SOM), self-organising map discriminant index (SOMDI) feature extraction and classification **(d)**. SKiNET is optimised on the training data using 10-fold cross validation and adjusting the number of neurons, the initial learning rate and the number of training steps to maximise the classification accuracy on the training data. Finally, the optimised model is shown the unseen test data and asked to classify each spectrum as either healthy, non-mildew covered, or mildew covered.

S2. Raman Spectra with Standard Deviation Shading:

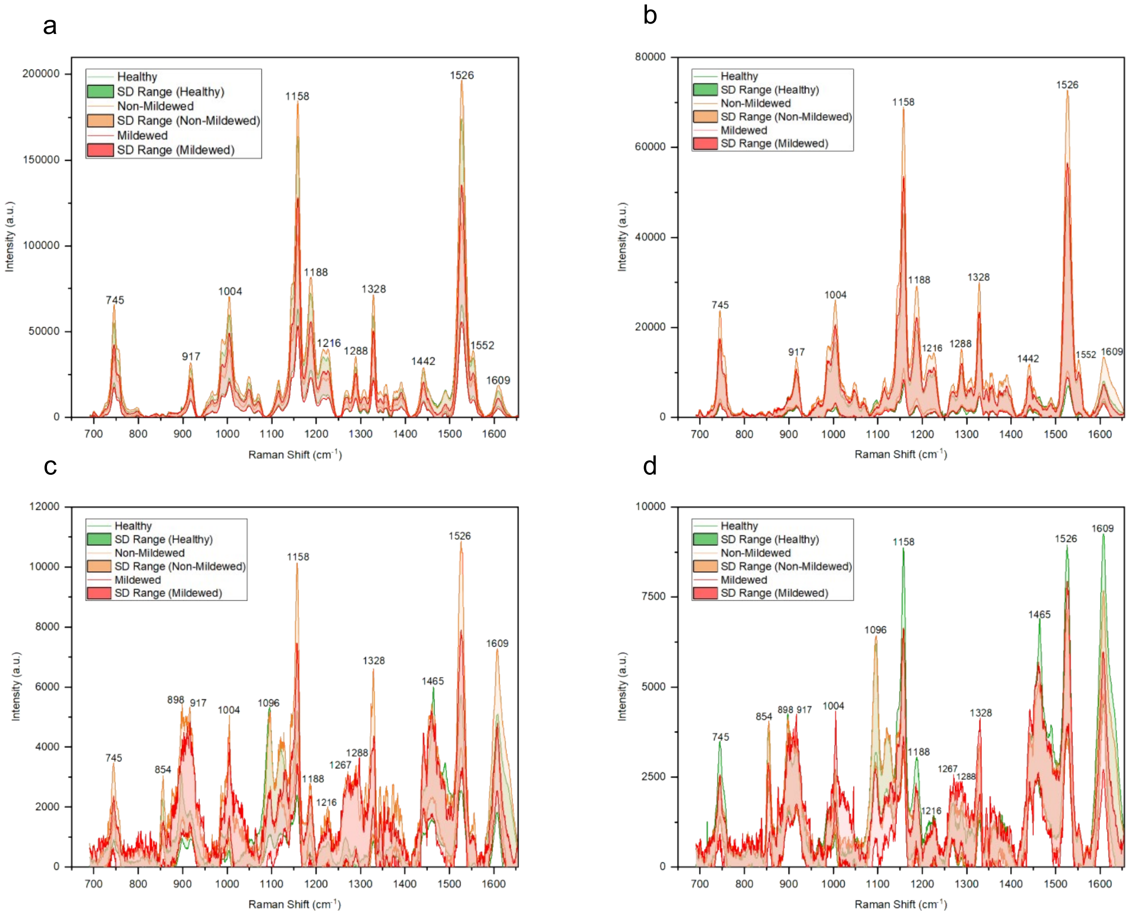

Figure S2. Raman spectra with standard deviation shading of the representative shift of the characteristic peaks present obtained from areas of **(a)** non-vein, **(b)** venule, **(c)** lateral vein and **(d)** mid-vein tissue types on the *Q. robur* leaf, which were either from a healthy leaf or an infected leaf, distinguishing between mildew-covered and non-mildew covered areas of infected leaves. Lines represent the minimum and maximum Raman signal intensities achieved by those respective disease classes on their respective tissue types.

S3. Non-Vein Intensity Range Box and Whisker Plot:

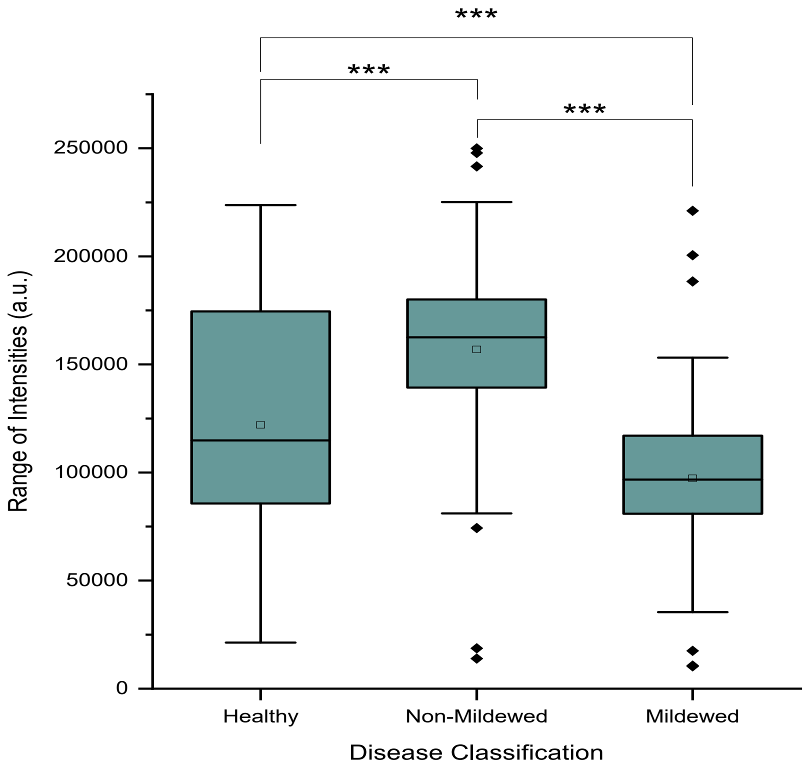

Figure S3. Box and whisker plot of the range of intensities from spectroscopic measurements of non-vein tissue from healthy, non-mildew covered and mildew-covered *Quercus robur* leaves. Brackets indicate significantly different pairs of ranges compared *via* a Mann-Whitney U test (p***<0.005).

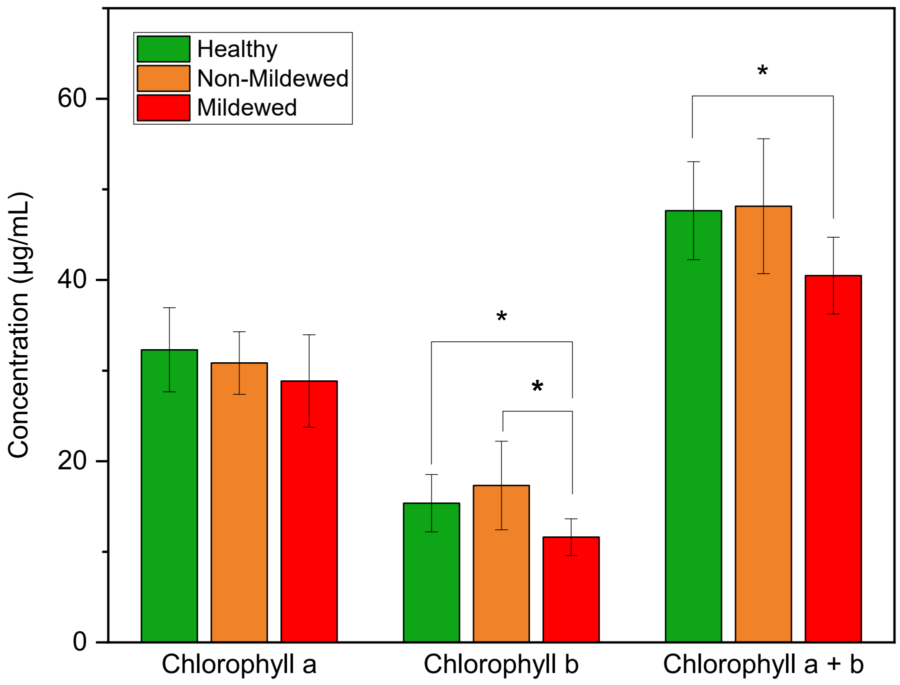

S4. Chlorophyll Concentrations:

Figure S4. Chlorophyll concentrations (µg/mL) from non-vein and venule containing tissues of *Quercus robur* leaves following extraction with 80% DMSO. Significantly different pairs of concentrations compared *via* a Mann-Whitney U test (p*<0.05) are highlighted.

S5. Reproducibility of Raman Spectroscopy Detection on Non-Vein Tissues from Healthy, Non-Mildew

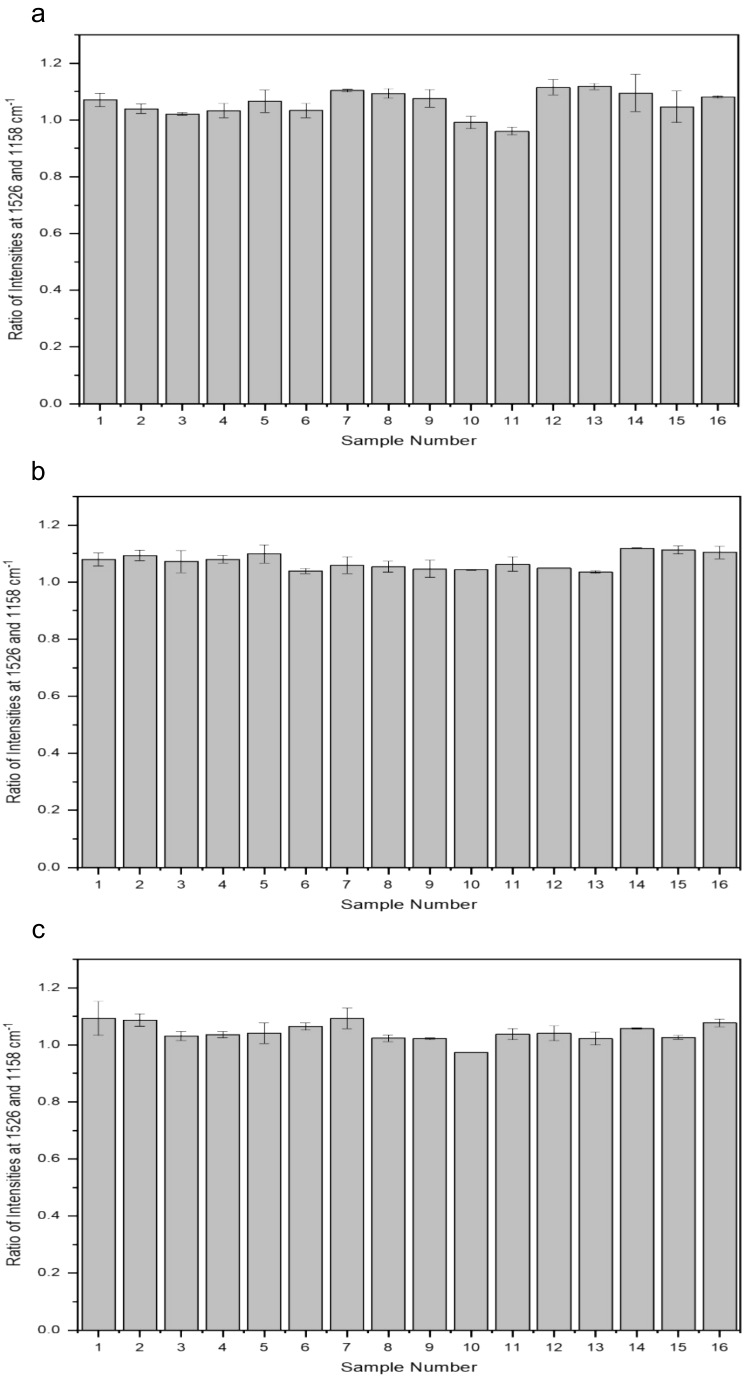

Covered and Mildew-Covered *Quercus robur* Leaves:

Figure S5. Variability of the ratio of the intensities at 1526 and 1158cm^{-1}, calculated as I(1526 cm^{-1}) / I(1158 cm^{-1}), where I(x cm^{-1}) is the intensity at x cm^{-1}, of non-vein tissue of **(a)** healthy, **(b)** non-mildew covered and **(c)** mildew-covered *Quercus robur* leaves.

Table S5.1: Summary of reproducibility coefficients (RCs) determined from **Fig. S5** calculated *via*: RC = standard deviation x 2.77 x 100, where 2.77 is chosen for a 95% level of confidence. Confidence interval limits (CILs) were calculated as: $CIL = \bar{r} \pm 1.96\sigma$, where $\bar{r}$ is the mean ratio and σ is the standard deviation.

Disease Class	Reproducibility Coefficient	Lower Confidence Interval Limit	Upper Confidence Interval Limit

Healthy	12.4%	0.972	1.15
Non-Mildew Covered	7.54%	1.02	1.13
Mildew Covered	8.83%	0.983	1.11
